# Long-term radiological assessment of a Mediterranean freshwater ecosystem surrounding a nuclear power plant

**DOI:** 10.1007/s11356-024-33140-0

**Published:** 2024-04-08

**Authors:** José Ángel Corbacho, Javier Guillén

**Affiliations:** 1 Departamento de Física Aplicada, Centro Universitario de Mérida. Universidad de Extremadura, 06800 Mérida, Badajoz Spain; 2 LARUEX, Laboratorio de Radiactividad Ambiental, Departamento de Física Aplicada, Facultad de Veterinaria, Universidad de Extremadura, Avda. Universidad, S/N 10003, Cáceres, Spain

**Keywords:** Non-human biota, Man-made radionuclides, Mediterranean ecosystem, Transfer coefficients, Dose modelling

## Abstract

The radionuclide concentration of man-made radionuclides on non-human biota in freshwater ecosystems has been extensively studied in environments affected by the Chernobyl and Fukushima accidents, in both humid continental and subtropical climates, respectively. However, there are very few studies that assess the long-term effects of operating nuclear facilities in Mediterranean environments. In the present study, a temporal analysis of the impact on carp, cattail, and bulrushes in the cooling pond of the currently operating Almaraz nuclear power plant was investigated for the period 2000–2020. The results do not show a general trend in man-made radionuclide concentrations. Instead, depending on their availability and the type of organism, trends decrease over time. This is also reflected in the effective half-lives obtained, which are lower than the physical half-life for some radionuclides. Transfer coefficients for the main man-made radionuclides detected were obtained, and it was found that these were significantly lower than the typical ranges found for benthic fish and vascular plants in freshwater ecosystems. Finally, the internal and external doses received by the carp have been evaluated using ERICA tool, and it has been observed that the main contribution to the total dose is due to the internal dose (0.65–7.04) × 10^−4^ µGy/h.

## Introduction

Radiological protection of the environment was previously considered adequate if humans were adequately protected from an anthropogenic point of view (ICRP 1991, 1997). In the case of freshwater ecosystems, data are often limited to regions affected by the deposition of radionuclides, as occurred in the Chernobyl or Fukushima accidents, or in the vicinity of nuclear power plants (NPPs) (Rowan and Rasmussen 1994; McCreedy et al. 1997; Sundbom et al. 2003; Smith et al. 2009; Wada et al. 2013), which are used for assessing the effective ingested dose to humans (Delistraty et al. 2010; Tjahaja et al. 2012). This perspective changed to ensure adequate protection of the environment itself (ICRP 2008a), with the introduction of the concept of Reference Animals and Plants (RAPs) (ICRP 2008b).

Since the 2000s, a significant number of studies have been published regarding long-term radionuclide concentration in aquatic wildlife due to routine radioactive discharges from NPPs. For example, the concentrations of tritium, ^60^Co, and ^137^Cs in different samples of Danube water, sediment, and various aquatic organisms collected upstream and downstream of the outlet of the warm water channel of the Paks Nuclear Power Plant (Hungary) were determined (Janovics et al. 2014). Similar studies have also been conducted in the Vltava River system in the Czech Republic (Hanslík et al. 2005, 2018); in the Loire River (France), where 14 nuclear power plants are located (Ciffroy et al. 2005); or in Lake Druksiai, which provided cooling water for the Ignalina NPP (Lithuania) until its closure in 2009 (Nedveckaite et al. 2011). There are also some studies focused on the bioaccumulation of man-made radionuclides in sea or freshwater food products originating from nuclear tests carried out in the 1950s–1960s (Aarkrog et al. 2000; Brittain and Bjørnstad 2010).

These studies provide the corresponding input to assess the exposure of non-human biota, expressed in terms of dose rate, using quasi-equilibrium models to estimate the activity concentration of the organisms and the internal and external doses, such as the ERICA Tool (Brown et al. 2008, 2016). One of the basic transfer parameters used in the models is the concentration ratio, CR_wo_, which relates the activity concentration in the organism (or RAPs where reported) and in the corresponding environmental media. These CR_wo_ values were compiled in a database described in Copplestone et al. (2013) (see http://www.wildlifetransferdatabase.org/), which was used to create compilations such as ICRP 114 (Annex A.1) (ICRP 2009) or IAEA Technical Report No. 479 (IAEA 2014). Although it has been updated (Brown et al. 2016), there are some limitations. For example, there are many RAP-element combinations lacking, some of the available data are highly site-specific and contribute to a large variability of the data, and there are biases in the available data (Wood et al. 2013). Most of the data in that online database come from Europe, Japan, North America, and Australasia and mainly from temperate and arctic ecosystems (Howard et al. 2013). Due to these biases, there is a scarcity of data for transfer parameters in Mediterranean ecosystems, such as Spain. The Mediterranean climate is characterized by very hot and dry summers with little rainfall during the summer period and humid winters in which rainfall is concentrated. In the case of terrestrial ecosystems, some data were reported in a previous work (Guillén et al. 2018). However, there is still a lack of data for freshwater ecosystems, including rivers and lakes, among other continental water bodies. In fact, the available data in IAEA TRS479 does not include data for freshwater ecosystems from Mediterranean countries (IAEA 2014). This lack of data is partly due to the low concentration of anthropogenic radionuclides in the environment, since fallout from both global nuclear weapons tests and the Chernobyl accident was comparatively low (de Cort et al. 1998).

The main goal of this work is to analyse the long-term radiological status of a freshwater ecosystem in a Mediterranean climate area, to estimate the corresponding transfer parameters, and to assess the dose rate to non-human biota. The selected area was the Arrocampo reservoir in the Tagus River, which is used for cooling purposes by the Almaraz Nuclear Power Plant which is located in the Southwest of Spain. Three different species of freshwater biota, carp (*Cyprinus carpio*), and two macrophyte species (bulrush *Scirpus* sp. and cattail *Typha latifolia*) were systematically sampled and analysed during the period 2000–2020. Time series were considered to derive effective half-lives. Finally, dose rates to non-human biota (carp) were assessed using the ERICA Tool.

## Material and methods

### Sampling site

**Fig. 1:**
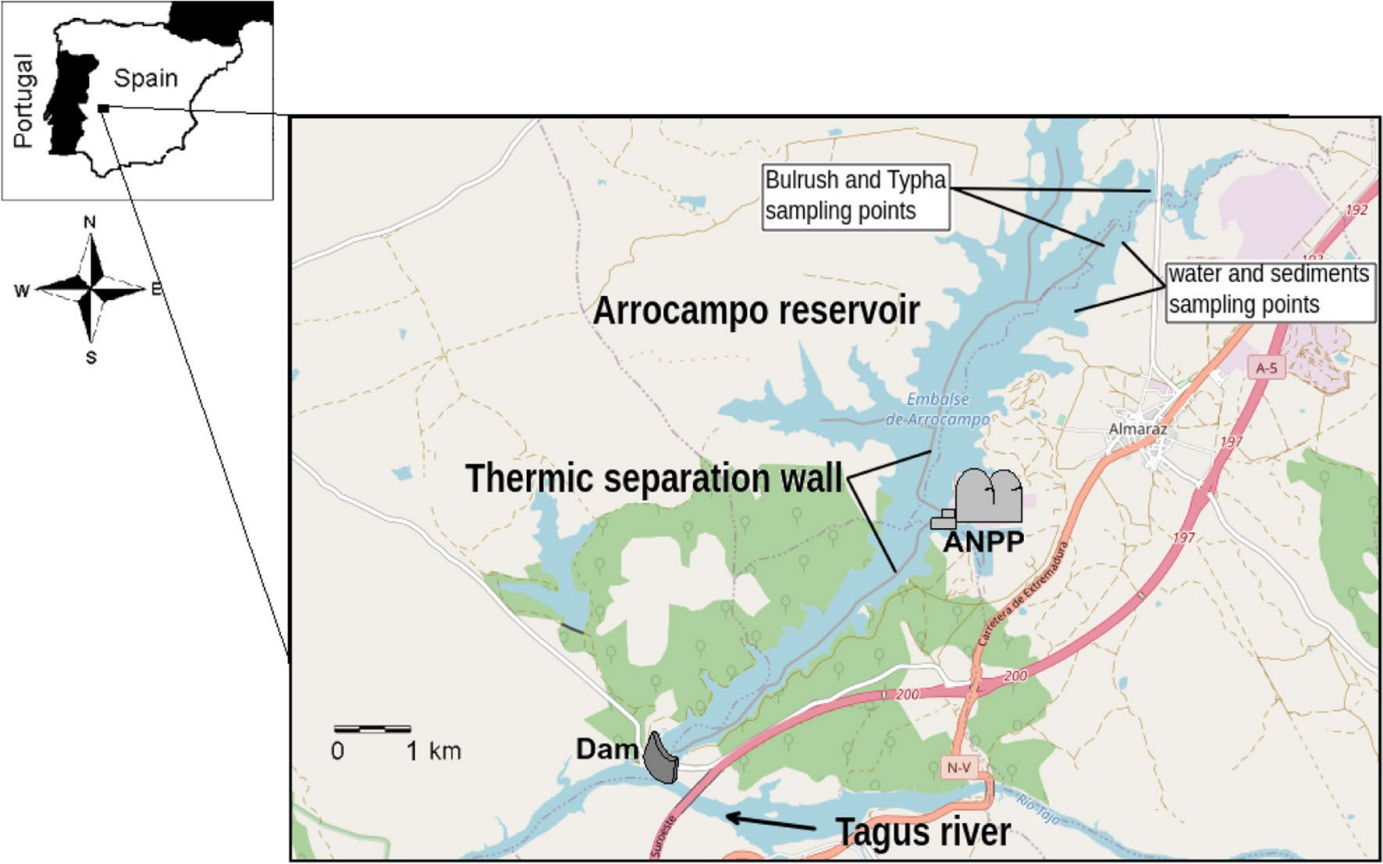
Scheme of Arrocampo reservoir, serving as secondary coolant for ANPP. Approximate locations of Bulrush and Cattail (*Scirpus* and *Typha*), sediments and water sampling points are shown (OpenStreetMap 2015)

This study was carried out in the Arrocampo reservoir (39°49′00″ N; 5°42′00″ W), which is located in the Extremadura region (southwest of Spain), close to the Almaraz Nuclear Power Plant (ANPP). The Arrocampo dam was built on the Arrocampo stream and its landform in 1976 to receive the ANPP’s warm water discharges from the secondary coolant circuit. To cool the ANPP’s steam turbines, water is taken from the Tagus River, discharged into the Arrocampo reservoir, flows through a U-shaped circuit of 25 km, passes through air-column coolers, and finally is discharged into the Tagus River (see Fig. 1). This process reduces the water temperature to environmental levels. To extend the length of the water circuit, a concrete thermal wall was built, which has a total length of 11 km and rises from the bottom of the reservoir to a height of 8 m, with 1 m of it being above the water surface. The surface area of the Arrocampo reservoir is approximately 8 km^2^. Due to the presence of a large number of wild birds, the Arrocampo reservoir is designated as a special wildlife protection area.

The climate of the region where the ANPP and Arrocampo reservoir are located is of the Mediterranean type. According to the Köppen climate classification, the region is classified as *Csa* “hot dry summer” (Kottek et al. 2006) (AEMET 2011). Regions classified as Csa, at least, in eight months must have average temperatures of 10 °C or higher, and the average annual precipitation must not exceed 900 mm. It is primarily characterized by very hot and dry summers with minimal rainfall during the summer season (precipitation less than 30 mm). Winters are relatively mild and prolonged (average annual temperature range: 10–29 °C (Felicísimo Pérez et al. 2001)), influenced by the proximity to the Portuguese Atlantic coast, which brings an oceanic influence. The majority of rainfall occurs during the winter months, with precipitation concentrated in that period. The annual precipitation average in the study area is 500–600 mm (Felicísimo Pérez et al. 2001).

To assess the radiological impact on the special ecosystem of Arrocampo wetland due to the routine operation of the ANPP, a monthly sampling campaign was undertaken from 2000 to 2020 at the same locations (see Fig. 1). The campaign involved collecting 20 L of surface water, 2 kg of sediments, and two or three individuals of European carp (*Cyprinus carpio*) as radiological bioindicators (purchased from local fishers). No samples were taken and analysed between June 2002 and December 2003.

Moreover, Directive 2000/60/EC (EU 2000) considers macrophytes as indicators of the ecological status of the water body. Therefore, two species of macrophytes, bulrush (*Scirpus*) and cattail (*Typha latifolia*), have been included in this study. The activity values of natural and man-made radionuclides in bulrush and cattail from 2006 to 2020 were extracted from the internet database of the Spanish Nuclear Safety Council (CSN 2023).

### Sample preparation

Carp samples were taken as part of the environmental monitoring plan of the nuclear power plant. Therefore, the skin and bone parts of the carp samples were removed, and only their muscle tissue was analysed. The samples were dried at 100 °C to remove water content and then reduced to ashes at 400 °C to reduce the volume. The samples were encapsulated in 191 cm^3^ capsules and measured using low-background gamma spectrometry. Subsequently, the biota samples were calcined at 600 °C to eliminate all organic matter for the radiochemical separation of ^90^Sr.

The sediment samples were dried at 100 °C to remove water content. Subsequently, they were placed in Marinelli beakers and sealed. The gamma spectrometry measurements were conducted after 28 days when ^226^Ra reaches its secular equilibrium with its daughters. For the determination of ^90^Sr, an aliquot of 50 g was calcined at 600 °C.

Water samples were evaporated, and the resulting dry residue was encapsulated in petri dishes for measurement using low background gamma spectrometry. Additionally, an aliquot of 5 L from each sample was separated specifically for the determination of ^90^Sr.

### Radionuclide determination

The γ-spectrometric analysis was performed using multiple p-type and extended energy germanium detectors. Each detector had a relative efficiency range of 37–60% and a resolution of 1.86 keV for the 1332 keV ^60^Co peak. The samples were systematically analysed for various man-made radionuclides, including ^51^Cr, ^54^Mn, ^58,60^Co, ^131^I, ^133,140^Ba, ^140^La, ^134,137^Cs, ^95^Zr, ^95^Nb, ^59^Fe, ^65^Zn, ^106^Ru, ^124,125^Sb, ^241^Am and ^110m^Ag. Additionally, the naturally occurring radionuclides ^40^ K, ^7^Be, and the ^238^U and ^232^Th series in equilibrium with their daughters were analysed.

The radiochemical method used for the determination of ^90^Sr in biota and water samples involved the retention of strontium as chloride using an anion resin, while preventing interference from calcium through the use of an EDTA solution (EML 1976). Subsequently, strontium was eluted with an aqueous solution of NaCl and precipitated as SrCO_3_ onto a steel planchet with a diameter of 5 cm. In the case of sediment samples, 50 g aliquots of the sediment were digested with acid, and strontium and barium were precipitated as nitrates. This precipitate was dissolved in distilled water, and interfering elements, primarily iron, were precipitated as hydroxides (Gascó and Álvarez 1988). Strontium was then precipitated as SrCO_3_ onto a 5-cm diameter steel planchet at pH 8. In both methods, the recovery yield was determined by gravimetry, using a known quantity of stable strontium as a carrier. After reaching equilibrium between ^90^Sr and ^90^Y (approximately 21 days), the samples were measured in a gas flow proportional counter.

The laboratory conducting the measurements maintains overall quality control through accreditation for performing radioactivity assays in environmental samples, in accordance with UNE-EN ISO/IEC 17025 (ISO 2017). To verify the quality of the measurements, various reference materials provided by the IAEA were used. These reference materials allowed for systematic confirmation of activity levels within the recommended intervals.

### Statistical analysis

#### Mann–Kendall trend test

The Mann–Kendall (M–K) test is a non-parametric statistical test used to identify trends in time series data. It is commonly employed to determine whether there is a statistically significant increasing or decreasing trend in long-term temporal data. The test is based on two hypotheses: the null hypothesis (H_0_) states that there is no trend, while the alternative hypothesis (H_1_) suggests the presence of a significant trend over a given time period.

In this test, each data value is compared to all subsequent data values. The Mann–Kendall statistic, denoted as *S*, is initially set to 0. If a data value from a later time period is greater than a data value from an earlier time period, *S* is incremented by 1. Conversely, if the first data value is lower than the earlier one, *S* is decremented by 1. The final value of *S* is determined by the net result of these increments and decrements.

The probability, also known as the *p*-value, is calculated based on the variance and the S statistical parameter. A significance level of 5% is commonly used. If the *p*-value is less than or equal to 0.05, the alternative hypothesis (H_1_) is accepted, indicating the presence of a trend. However, if the *p*-value is greater than 0.05, the null hypothesis (H_0_) is accepted, indicating no trend in the data.

#### Shapiro–Wilk test

The Shapiro–Wilk (S-W) test is a statistical test used to assess whether a sample of data follows a normal or lognormal distribution. It can be applied to evaluate these distributions separately. The null hypothesis for this test states that the data sample is derived from a normally or lognormally distributed population. If the *p*-value is less than 0.05 (at a 5% significance level), the null hypothesis is rejected, providing evidence that the tested data does not follow a normal or lognormal distribution.

In this study, the Shapiro–Wilk test was employed to examine the normality of the frequency distributions of the activities. If the test result yields a negative result (*p*-value less than 0.05), indicating non-normality, the Shapiro–Wilk test is further applied to determine whether the frequency distribution corresponds to a lognormal distribution. If the result is once again negative, it indicates that the population of analysed data exhibits a frequency distribution that is neither normal nor lognormal.

### Transfer coefficients and dose assessment to non-human biota

Transfer parameters in this freshwater ecosystem for carps, CR_wo-water_, bulrush, CR_wo-sediment_, and apparent *K*_*d*_ values were calculated according Eqs. 1–3 (IAEA 2014). Apparent *K*_*d*_ is not properly determined by absorption or de-sorption methodology but can be considered as a ratio under quasi-equilibrium conditions, where the contact time of water and sediment is very large.

These parameters were calculated only when there was activity concentration above detection limit in the two compartments involved.

1 $${\text{CR}}_{{\text{wo}}-{\text{water}}}\text{=}\frac{{\text{C}}_{\text{carp}}}{{\text{C}}_{\text{water}}}\cdot{\text{Ratio}}_{\text{wo:muscle}} \,$$

2 $${\text{CR}}_{\text{wo-sediment}}\text{=}\frac{{\text{C}}_{{\text{bulrush}}/{\text{cattail}}}}{{\text{C}}_{\text{sediment}}} \,$$

3 $${\text{Apparent}}{\text{ K}}_{\text{d}}\text{=}\frac{{\text{C}}_{\text{water}}}{{\text{C}}_{\text{sediment}}} \,$$

where *C*_carp_ is the concentration in carp muscle, expressed in Bq/kg fw; *C*_bulrush/cattail_ is the concentration in bulrush or cattail, expressed in Bq/kg fw; *C*_water_ is the concentration in surface water, expressed in Bq/L; *C*_sediment_ is the concentration in sediment, expressed in Bq/kg dw; and Ratio_wo:muscle_ was conversion factor for muscle to whole organism from IAEA TRS 479 (IAEA 2014).

Dose assessment for non-human biota (carp) was evaluated using Tier 3 in ERICA Tool 2.0 (Brown et al. 2008, 2016), using probabilistic data from the experimental results (radionuclide concentrations, concentration ratios and apparent *K*_*d*_ values) with 10,000 simulations. Due to the nature of this study, Tiers 1 and 2 have been omitted. As radionuclide concentration in fish was determined in muscle tissue, the Ratio_wo:muscle_ defines ratio between whole organism concentration and that of a given tissue (muscle in this case). For freshwater fish, it was reported to be 1 for Cs, Co, and Mn; 38 for Sr; and 2.1 for Zn (Yankovich et al. 2010; IAEA 2014). The predefined organism pelagic fish was used to assess internal and external dose rates to carps. Weighted dose rates were estimated using the default radiation weighting factors from the ERICA Tool of 10 for *α*, 3 for low energy *β* and 1 for other *β* and *γ* emissions.

### Determination of effective and ecological half-lives

The effective half-life is the time taken for the amount of radioactivity in an environmental component to reduce by one half following exposure in the natural environment and therefore includes declines due to the physical decay of the radionuclide (Smith and Beresford 2005).

In order to obtain the effective half-life (*T*_eff_), the monthly concentrations of the radionuclides were analysed by using the following regression equation:

4 $${\text{ln}}{C}_{j}=-{\lambda }_{{\text{eff}}}\cdot t+{\text{ln}}{ C}_{0}$$

where *C*_*j*_ is monthly activity concentration in month *j*, *λ*_eff_ is the effective rate of decline in radioactivity concentration, calculated as the slope (1/m), *t* is time of the sampling (month) and *C*_0_ is initial concentration at the beginning of the sample campaign.

The effective half-lives (*T*_eff_) were calculated from the decrease in radionuclide activity according to the equations (Smith and Beresford 2005).

5 $${T}_{{\text{eff}}}=\frac{{\text{ln}}2}{{\lambda }_{{\text{eff}}}}$$

The presence and availability of a radionuclide to biota depend on its physical and ecological half-lives, which is determined by excretion (biological elimination from the living body) and influenced by ecological factors such as abiotic factors (leaching and immobilisation of the nuclide in water and sediments, etc.). The effective half-life combines both the physical and ecological decays. As a result, the inverse of effective half-life is the sum of the inverses of the physical and ecological half-lives:

6 $$\frac{1}{{T}_{{\text{eff}}}}=\frac{1}{{T}_{{\text{phys}}}}+\frac{1}{{T}_{{\text{ecol}}}}$$

If the object is not a biological organism, *T*_ecol_ is not used. Instead, the environmental half-life (*T*_env_) is used because an abiotic compartment does not itself have a biological factor (Tagami and Uchida 2016).

It is important to take account that according to Eq. 6, the effective half-life of a radionuclide in an ecosystem is always shorter than the physical half-life, provided that no additional influx of radionuclides occurs to the ecosystem.

## Result and discussion

### Radionuclide concentration in freshwater ecosystem

**Fig. 2:**
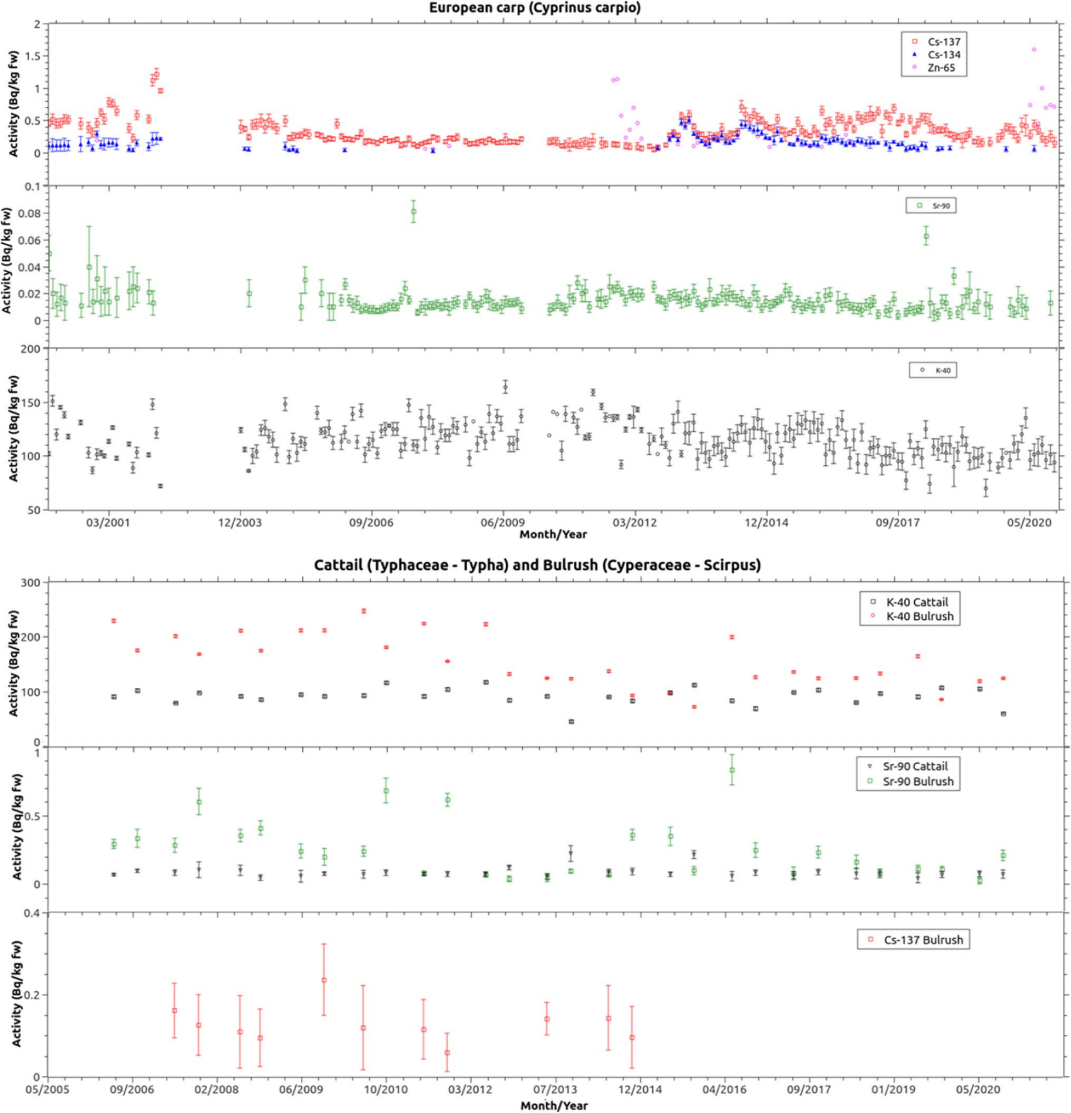
Representation of the activity levels and uncertainties of ^40^ K, ^137^Cs, ^134^Cs and ^90^Sr with activity greater than the minimum detectable activity (MDA) measured in carp (*Cyprinus carpio*), bulrush (*Scirpus*), and cattail (*Typha*) samples

**Fig. 3:**
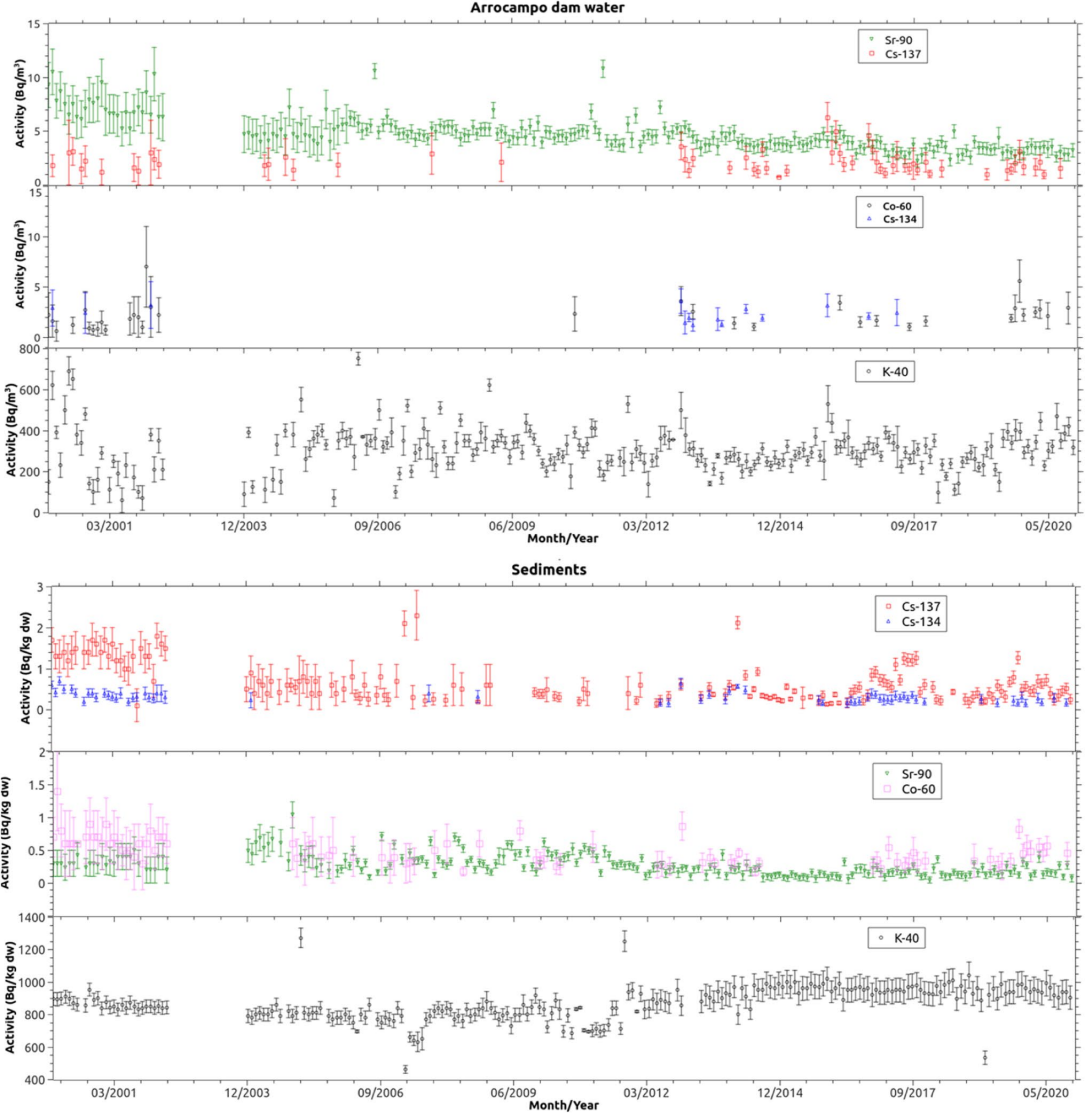
Representation of the activity levels and uncertainties of ^40^ K, ^137^Cs, ^134^Cs, ^60^Co and ^90^Sr with activity greater than the minimum detectable activity (MDA) measured in sediment and surface water samples

Figures 2 and 3 display the activity concentrations of natural and man-made radionuclides measured in carp, bulrush, cattail, water, and sediment samples from 2000 to 2020. The figures only depict the activity concentrations that were detected in more than 10 samples throughout the entire study period.

Table 1 shows the arithmetic mean, standard deviation, median and, when the number of samples with activity greater than MDA was greater than 10, the geometric mean, geometric standard deviation and a basic characterisation of the frequency distribution (skewness, kurtosis, and Saphiro-Wilk test) for the organic samples analysed for the period 2000–2020 (carp) and for the period 2006–2020 (bulrush and cattail). Concerning carp samples, ^40^ K, ^137^Cs and ^90^Sr were detected in almost all samples, while ^134^Cs was detected in 46%, ^65^Zn in 8.9% and occasionally ^54^Mn, ^59^Fe,^110m^Ag and ^60^Co. In terms of activity concentration, the mean values and range of man-made radionuclides are of the same order of magnitude as those reported at the Handford site on and off site (Delistraty et al. 2010) and lower than some values reported in the literature (Rowan and Rasmussen 1994; Nedveckaite et al. 2011). All of them involved the analysis of the whole fish, unlike this study, in which fishbone and skin were removed prior to radionuclide determination. For bulrush and cattail, the radionuclides ^40^ K and ^90^Sr were detected in all samples, while ^137^Cs was detected in 36.6% of bulrush samples. Other man-made radionuclides such as ^65^Zn, ^60^Co, and ^134^Cs were below the MDA. For ^65^Zn, the MDA is on the order of 0.2 Bq/kg, and for the other radionuclides, it is below this value.

**Table 1:** Basic statistics of natural and anthropogenic activity values measured in organic samples (carp, cattail and bulrush)

	Average ± SD	Range	*N*	(1)	Median	Geometric mean (GMSD)	Frequency distribution shape			Trend M–K test
							Skew	Kurt	S-W test	
Carps (muscle part Bq/kg, fresh weight)										
^90^Sr	0.015 ± 0.009	0.004–0.081	211	180	0.014	0.013 (0.021)	1.759	22.24	*Logn*	▼
^137^Cs	0.33 ± 0.20	0.046–1.22		208	0.282	0.281 (0.493)	1.395	3.42	*Logn*	–
^134^Cs	0.17 ± 0.10	0.055–0.503		97	0.15	0.145 (0.267)	1.245	1.59	*Not*	–
^60^Co	0.06 ± 0.04	0.029–0.139		8	0.061	0.054 (0.089)	–	–	–	–
^65^Zn	0.4 ± 0.4	0.086–1.6		19	0.169	0.292 (0.719)	1.608	2.83	*Not*	–
^59^Fe	0.33 ± 0.13	0.201–0.451		3	0.322	–	–	–	–	–
^54^Mn	0.059 ± 0.007	0.058–0.059		2	–	–	–	–	–	–
^40^K	120 ± 20	70–164		211	113	113.9 (131.7)	0.128	− 0.04	*Normal*	▼
Bulrush (*Scirpus*) (Bq/kg, fresh weight)										
^90^Sr	0.25 ± 0.20	0.025–0.836	30	30	0.221	0.177 (0.437)	1.274	1.217	*Logn*	▼
^137^Cs	0.13 ± 0.5	0.059–0.236		11	0.119	0.12 (0.17)	1.224	2.929	*Logn*	–
^134^Cs	0.090 ± 0.001	0.09–0.1		2	–	–	–	–	–	–
^40^K	160 ± 50	72–247		30	146	150 (206)	0.164	− 1.029	*Normal*	▼
Cattail (*Typha*) (Bq/kg, fresh weight)										
^90^Sr	0.09 ± 0.04	0.044–0.230	30	30	0.074	0.080 (0.113)	2.674	7.564	*Logn*	–
^40^K	90 ± 20	45–117		30	92	90 (110)	− 0.997	2.033	*Normal*	–

*N* < 10 frequency distribution and M–K test have not been calculated

*GMSD* geometric standard deviation; *N* number of samples analysed; (1) samples with activity > MDA (minimum detectable activity); *S-W test* Saphiro-Wilk test (*p*-value is less than < 0.05 (5% significance level). *Normal or Logn p*-value is greater than 0.05; the data set is from a normally or lognormally distributed population); *Trend M–K test* Mann–Kendall trend test

^▼^Statistically significant evidence of a decreasing trend at the specified level of significance (0.05)

^▲^Statistically significant evidence of an increasing trend at the specified level of significance (0.05)

The radionuclides ^40^ K and ^90^Sr were detected in all surface water and sediment samples analysed, while ^137^Cs was detected in 26.9% of surface water samples and 73.3% of sediment samples. The radionuclides ^60^Co and ^134^Cs were detected in 15.7% and 6.2% of the surface water samples, respectively; ^134^Cs and ^60^Co were detected in 40.4% and 29.7% of the sediment samples. Activity concentrations of ^137^Cs and ^90^Sr were similar to those reported for rivers or lakes affected by discharges from operating NPPs (Rowan and Rasmussen 1994; Aarkrog et al. 2000; Hanslík et al. 2018; Duffa et al. 2004).

The frequency distribution of the activity of natural radionuclides is usually of normal or Gaussian type, whereas the distribution of radionuclides of artificial origin follows a lognormal distribution, especially if there are contributions to the environment from these radionuclides. In this sense, as shown in Table 1 and 2, the Saphiro-Wilk tests show that radionuclides of natural origin (^40^ K) show a normal distribution in the organic samples (carp, bulrush and cattail) as expected because [K] is self-regulated in living bodies.

**Table 2:** Basic statistics of natural and anthropogenic activity values measured in surface water and sediments samples

	Average ± SD	Range	*N*	(1)	Median	Geometric mean (GMSD)	Frequency distribution shape			Trend M–K test
							Skew	Kurt	S-W test	
Arrocampo sediments (Bq/kg, dry weight)										
^90^Sr	0.28 ± 0.17	0.046–1.100	227	223	0.240	0.233 (0.422)	1.511	3.83	*Logn*	▼
^137^Cs	0.70 ± 050	0.10–2.30		165	0.50	0.533 (1.035)	1.293	1.07	*Logn*	–
^134^Cs	0.31 ± 0.12	0.16–0.70		67	0.29	0.292 (0.423)	1.050	1.07	*Logn*	–
^60^Co	0.5 ± 0.2	0.17–1.40		91	0.45	0.445 (0.662)	1.359	2.48	–	▼
^40^ K	900 ± 100	463–1270		225	870	863 (973)	− 0.42	− 0.42	*Not*	–
Surface water (Bq/m^3^)										
^90^Sr	4.7 ± 1.5	2.2–10.8	231	231	4.5	4.51 (6.032)	1.465	2.98	*Not*	▼
^137^Cs	2.1 ± 1.0	0.8–6.3		60	1.9	1.939 (2.917)	1.847	4.76	*Logn*	–
^134^Cs	2.3 ± 0.8	1.2–3.6		14	2.3	2.217 (3.038)	0.150	− 1.06	*Not*	–
^60^Co	2.1 ± 1.3	0.6–7.0		35	2.0	1.828 (3.223)	1.951	5.32	*Logn*	▲
^58^Co	4.6 ± 4.2	1.3–11.8		5	3.2	–	–	–	–	–
^40^ K	300 ± 100	60–750		223	297	282 (420)	0.732	2.15	*Not*	–

*N* < 10 frequency distribution and M–K test have not been calculated

*GMSD* geometric standard deviation; *N* number of samples analysed; (1) samples with activity > MDA (minimum detectable activity); *S-W test* Saphiro-Wilk test (*p*-value is less than < 0.05 (5% significance level). *Normal or Logn p*-value is greater than 0.05, the data set is from a normally or lognormally distributed population); *Trend M–K test* Mann–Kendall trend test

^▼^Statistically significant evidence of a decreasing trend at the specified level of significance (0.05)

^▲^Statistically significant evidence of an increasing trend at the specified level of significance (0.05)

Regarding man-made radionuclides, the concentration in both living organisms and in water or sediments is due, among other factors, to the amount injected into the ecosystem and how this amount is distributed in the ecosystem. Therefore, it is unusual for the frequency distribution for artificial radionuclides to be Gaussian. In this sense, as expected, the frequency distributions of the radionuclides ^137^Cs, ^134^Cs and ^90^Sr in the organic samples are not Gaussian or normal, but of the lognormal type. In fact, as shown by the skewness coefficients obtained and the Saphiro_Wilk test applied for some of the man-made radionuclides detected in the organic samples, the frequency distribution of their activities per Bq/kg fw is lognormal.

Two primary sources of anthropogenic radionuclides justify their presence in the Arrocampo reservoir ecosystem: (a) past atmospheric nuclear tests conducted in the 1950s and 1960s (global fallout) and (b) liquid effluents released from the ANPP.

As it can be seen in Figs. 2 and 3, the activity levels of some radionuclides show a trend. However, it is not easy to visualise this. For this reason, in Table 1 and 2, the right column shows the result of the application of the Mann–Kendall test, which, based on a null hypothesis test with a significance level of 0.05, allows ensuring a statistically significant trend. This is why a decreasing trend can be observed in activity levels of ^90^Sr in carp and bulrush samples. In this case, the contribution from ANPP authorized discharges can be considered as negligible versus the global fallout contribution and the downward trend shows the disintegration of ^90^Sr.

Although ^40^ K concentration is self-regulated in living bodies (which is under strict homeostatic control in animals), therefore it shows very small variation regardless of variation in environmental levels. Activity concentrations for ^40^ K in carps at Arrocampo reservoir were within the range 70 to 164 Bq/kg fw; this agrees with literature (Hosseini et al. 2010). However, the M–K test shows a significantly statistically trend (slope − 0.0747; intercept: 123 Bq/kg), which might be due to a different parameter of the analysed carp samples. As Metz et al. (2003) describe, temperature strongly influences all biochemical processes in carps, and when a set of crucial physiological processes is subjected to changes in water temperature, internal homeostasis may become affected. Being a eurythermal fish, carp must have adaptive mechanisms to control internal homeostasis over a broad range of ambient temperatures. In fact, the concentration of ^40^ K in the water samples is more variable than one would expect.

Regarding the trend of ^137^Cs concentrations in biota, it can be observed in Table 1 that it is not possible to statistically affirm the presence of an increasing or decreasing trend from 2000 to 2020 in carps, nor from 2006 to 2020 in bulrush and cattail. However, Fig. 2 shows that the concentration of ^137^Cs in carps follows a decreasing trend between the period of 2000–2012. In 2012, a significant increase in ^137^Cs concentration is observed in the carp samples, along with the appearance of ^134^Cs. However, between 2012 and 2020, there is no noticeable trend in ^137^Cs concentration, although there is one for ^134^Cs. Separate statistical analyses of both periods and for both radionuclides demonstrate, as expected, that such trends are statistically significant with a confidence level of 95% for ^137^Cs concentration between the period of 2000–2012 and for ^134^Cs between the period of 2012–2020. From 2012 onwards, the presence of radiocaesium concentrations with activity higher than MDA in water samples is more frequent, indicating a higher bioavailability of radiocaesium, which is reflected in the concentrations in carp muscles samples but not in bulrush and cattail samples.

Unlike the trends observed in carps, the trend analysis of the activity concentration of naturally and man-made radionuclides measured in water and sediments does not provide a significant statistical result as can be shown in Fig. 2. Only the ^90^Sr activity concentration both in water and sediments shows a statistically significant trend that is expected because, as it was cited in previous section, the main ^90^Sr source in water and sediments is the former nuclear atmospheric test. However, regarding the concentration of ^60^Co in water and sediment samples, an interesting phenomenon is observed. The concentration of ^60^Co in sediment samples shows a statistically significant decreasing trend. Regarding the concentration of ^60^Co in water samples, it shows an increasing trend. There are occasional contributions of ^60^Co from routine discharges from the ANPP, which are reflected in the water samples. Due to the short residence time of ^60^Co in solution (Cornett and Ophel 1986), it is deposited in the sediments where its concentration decreases over time, but at a slower rate than its physical half-life, probably due to several routine discharges over the study period. Finally, this trend does not reflect in the specific activity detected in the analysed biota samples.

### Effective and ecological half-lives of man-made radionuclides in biota, water and sediment samples

Table 3 shows the effective and ecological half-lives values for the biota samples analysed, as well as the effective and environmental half-lives for water and sediment samples.

**Table 3:** Effective, ecological and environmental half-lives for the radionuclides ^90^Sr, ^134,137^Cs and ^60^Co in biota, water and sediment samples collected at Arrocampo dam

Sample	Radionuclide	Period	*T*_eff_ (y)^1^	*T*_ecol_ (y)
Carp	^90^Sr	(2000–2020)	30 ± 8	–
	^137^Cs	(2000–2012)	4.5 ± 0.2	5.6 ± 0.3
		(2012–2020)	17 ± 7	38 ± 15
	^134^Cs	(2000–2012)	6 ± 2	–
		(2012–2020)	3 ± 0.5	–
Bulrush	^90^Sr	(2006–2020)	8 ± 3	11 ± 4
	^137^Cs	(2006–2020)	16 ± 2	34 ± 4
Cattail	^90^Sr	(2006–2020)	70 ± 60	–
Sample	Radionuclide	Period	*T*_eff_ (y)^1^	*T*_env_ (y)
Water	^90^Sr	(2000–2020)	17 ± 1	45 ± 2
	^137^Cs	(2000–2020)	> 165	–
		(2000–2012)	> 150	–
		(2012–2020)	16 ± 11	30 ± 20
Sediments	^90^Sr	(2000–2020)	12 ± 1	20 ± 2
	^137^Cs	(2000–2020)	15 ± 2	32 ± 5
		(2000–2012)	5.2 ± 0.6	6 ± 1
		(2012–2020)	30 ± 30	–
	^134^Cs	(2000–2020)	24 ± 12	–
	^60^Co	(2000–2020)	25 ± 5	–

If the effective half-life (*T*_eff_) is greater than the physical half-life (*T*_phys_), the ecological (*T*_ecol_) or environmental (*T*_env_) half-life is not calculated.

It is important to note that the effective half-lives for ^137^Cs and ^134^Cs have been calculated in two separate periods for carp (2000–2012 and 2012–2020). As can be seen in Fig. 2, there is a significant increase and change in trend in the concentration of these two radionuclides at the end of 2012. Something less striking is also observed for the sediments and water of the Arrocampo reservoir. For this reason, the calculation of the effective half-lives was carried out for the whole period (2000–2020) and, also, for both periods (2000–2012 and 2012–2020).

First, many studies of effective half-lives have been carried out in environments directly affected by the Chernobyl and Fukushima nuclear accidents. However, there are fewer publications on ecosystems affected by controlled releases from nuclear facilities and the presence of global fallout from nuclear testing in the 1950s and 1960s. In addition, the variability of the long-term behaviour of ^137^Cs and ^90^Sr in freshwater ecosystems is much more pronounced than in terrestrial systems. It is strongly dependent on site-specific characteristics. The observed effective half-lives for ^137^Cs and ^90^Sr cover a wide range from several days to several years (Proehl et al. 2004).

It is worth noting that the effective half-life of ^90^Sr in carp is different from that observed in the rest of the biota samples analysed. The value is the same as the physical half-life and, therefore, the concentration of ^90^Sr in carp muscle decreases following the physical decay of this radionuclide (28.91 y). As already mentioned, the presence of ^90^Sr in the study area is mainly due to global fallout. In addition, the bioaccumulation of ^90^Sr in fish is influenced by many physical, chemical, biological and ecological factors, including ^90^Sr deposition, fish feeding habits, fish weight, trophic characteristics of the reservoir, calcium concentration in the water and other chemical and hydrological characteristics of the reservoir, as well as the quality of its drainage basin (Arrocampo stream). The values obtained are higher than those observed in similar studies that are between 7 and 17 years (Hanslík et al. 2005, 2018; Aarkrog et al. 2000).

With regard to ^137^Cs, the effective half-life in carp muscle obtained in this study is similar to the other works where studies were conducted in areas not significantly affected by the Chernobyl accident, the effective half-life values of ^137^Cs in fish range from 4 to 30 years (Franić and Marović 2007; Jannik et al. 2013; Paller et al. 2014; Brittain and Bjørnstad 2010; AMAP 2010). It is even similar to the effective half-life of ^137^Cs found in seawater fish in areas affected by nuclear testing in the past (Noshkin et al. 1997). However, it is important to note that the ecological half-life is of the order of 6 years in the period 2000–2012, whereas it increases by a factor of 6 in the period 2012–2020. This shows that the ^137^Cs contributed from 2012 onwards is more biologically available and therefore takes longer to be excreted by the carp.

Regarding flora samples, there is a significant difference between the ^90^Sr effective half-life in cattail and bulrush. On the one hand, the effective half-life of ^90^Sr in bulrush is lower by a factor of 10 than in cattail. This may be due to the nature of the plant, as both grow in semi-submerged and similar sediments where the availability of ^90^Sr should be the same.

The effective half-life of ^137^Cs in the bulrush is also lower than the physical half-life, so that the ecological half-life is slightly longer and of the order of the physical half-life. This is consistent with the result obtained for ^90^Sr, where it was indicated that, due to the nature of the bulrush, there is a decrease in the concentration of Sr and Cs.

In contrast to biota samples, where there is a biological elimination factor for the incorporated man-made radionuclides, in abiotic samples, the environmental half-life takes into account other parameters such as the precipitation of radionuclides in solution in the case of inland waters or the greater depth percolation of radionuclides in sediments.

For water samples, the effective half-lives are within the range (1–20 years) obtained by other authors on samples collected mainly from sites not directly affected by Chernobyl (Aarkrog et al. 2000; Hanslík et al. 2018). Similarly, for sediments, the effective periods of ^90^Sr and ^137^Cs are consistent with those obtained in similar studies conducted between 1993 and 2016 on sediment samples taken from the Orlík reservoir, which is affected by the Temelín nuclear power plant (Hanslík et al. 2018).

The effective half-lives of ^90^Sr obtained from the analysis of water and sediment samples show, as expected, given that the contribution is mainly from global fallout, that there is a slow environmental decrease in the concentration of this radionuclide in the Arrocampo ecosystem.

On the other hand, the effective half-life of ^137^Cs in water samples over the whole period studied (2000–2020) shows that there are at least several new inputs of ^137^Cs other than the first one considered (year 2000) and, as shown in Fig. 3, it corresponds to 2012. If only the period 2012–2020 is considered, an environmental half-life value of the order of the physical half-life of ^137^Cs (30.08 y) is observed, indicating a slow decrease in the concentration of ^137^Cs in the latter period compared to the first one, for which it was not possible to obtain an environmental half-life. With regard to the effective half-lives of ^137^Cs in sediments, in the two time periods studied, it is observed that in the period 2000 to 2012 it is a factor 6 lower than that obtained in the period 2012–2020 where the new contributions of ^137^Cs to the Arrocampo ecosystem are observed. Taking into consideration the entire study period, the environmental half-life is of the order of the physical half-life of ^137^Cs.

Finally, for the radionuclides ^134^Cs and ^60^Co, the effective half-lives determined in the sediment samples clearly indicate that there are several inputs over the whole study period (2000–2020).

Something quite striking in the whole study is the presence of ^134^Cs and ^137^Cs in carp muscles measured in the period 2012 to 2020. Figure 2 shows that there is a moment when the concentration of ^137^Cs increases and at the same time the concentration of ^134^Cs is detected. In order to calculate the experimental relationship ratio ^134^Cs/^137^Cs, *R*(*t*), the following expression was fitted to the experimental data:

7 $$R\left(t\right)={R}_{0}\cdot {\text{exp}}\left(C\cdot t\right);C={\text{ln}}\left(2\right)\cdot \left(\frac{1}{{T}_{{\text{Cs}}-137}}-\frac{1}{{T}_{{\text{Cs}}-134}}\right)$$

where *R*_0_ is the initial ratio at the moment when ^134^Cs activity is detected and *T*_Cs-137_ and *T*_Cs-134_ are the experimental half-life of ^137^Cs and ^134^Cs.

**Fig. 4:**
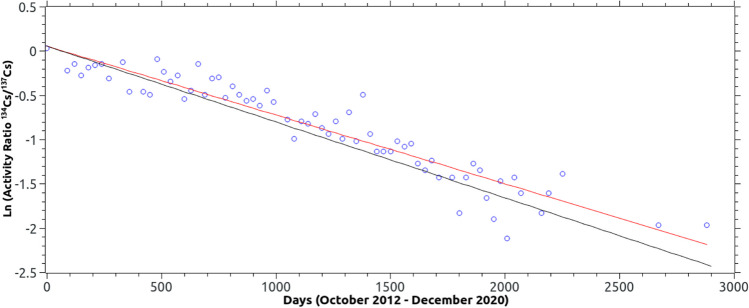
Natural logarithm of ^134^Cs/^137^Cs activity ratio. Black line: physical ratio. Red line: experimental ratio

Figure 4 shows the time evolution of the natural logarithm of the activity ratio of ^134^Cs/^137^Cs between the end of 2012 and 2020. A black line shows the graphical representation of Eq. 6 with the physical values of the half-lives of ^134^Cs and ^137^Cs and the value of *R*_0_ obtained at the time when ^134^Cs activity greater than MDA is detected, corresponding to the carp sample taken in October 2012: Act(^134^Cs): 0.075 ± 0.029 Bq/kg fw; Act(^137^Cs): 0.073 ± 0.043 Bq/kg fw; *R*_0_ = 1.0 ± 0.7. The theoretical value of the constant *C* from Eq. 7, using the physical half-lives of ^134^Cs and ^137^Cs, is − 8.56 ·10^−4^ (d^−1^).

The red colour shows the graphical representation of Eq. (7) after the linear fit to the experimental data of Ln *R*(*t*). The results obtained are *R*_0_ = 1.1 ± 0.9; *C* = -(7.8 ± 0.3) · 10^−4^ (d^−1^). As can be seen, the experimental value of C is 10% lower than the theoretical value. This result is consistent with the fact that ^134^Cs is incorporated into the carp population of the Arrocampo dam at a given time, when the routine discharge of the plant is carried out, and its presence in the carp decreases with its physical half-life. In fact, the result is consistent with the effective half-life obtained (Table 3).

### Transfer coefficients and radiological assessment for non-human biota in freshwater ecosystem

Table 4 shows the experimental values of CR_wo_water_ were compared with those found in IAEA report 479 (IAEA 2014). Therefore, carp is considered a benthic feeding fish and bulrush and cattail (*scirpus/typha*), which is not specifically cited in that report, is considered a vascular plant. The geometric mean of CR_wo_water_ for carps were slightly lower than the reported geometric mean value for Sr and Cs, but within the reported range. This difference may be due to the scarcity of transfer value data for Mediterranean ecosystems, as noted in previous studies (Guillén et al. 2018).

**Table 4:** Comparison of CR_wo_water_ and CR_wo_sediment_ values for ^40^ K, ^90^Sr, and ^134,137^Cs reported in this work and those reported for the corresponding elements for fish with benthic feeding and vascular plants in IAEA TRS 479 (IAEA 2014)

Radionuclide	CR_wo_water_ (freshwater fish)				
	This work (carp)			IAEA TRS 479 (fish benthic feeding)	
	*N*	GM	Range	GM ± GMSD	Range
^40^ K	201	403	170–1800	–	–
^90^Sr	172	114	36–600	330 ± 5	3.8–4.8·10^4^
^137^Cs	47	199	54–570	460 ± 4	18–2·10^4^
^134^Cs	10	103	45–400		
Radionuclide	CR_wo_sediment_ (vascular plant)				
	This work (bulrush/cattail)			IAEA TRS 479	
	*N*	GM	Range	GM ± SD	Range
^90^Sr (both)	30/30	0.8 / 0.36	0.15–7.21/0.1–1.54	61 ± 4	17–4400
^137^Cs (bulrush)	11	0.48	0.14–1.24	70 ± 6	17–2.4·10^4^
^134^Cs (bulrush)	2	0.32	0.24–0.44		

*N* number of samples, *GM* geometric median, *GMSD* geometric standard deviation

Table 5 shows the experimental sediment–water transfer coefficients, *K*_*D*_, obtained from the measurements made in this study. It is important to note that these apparent *K*_*D*_ coefficients have been calculated assuming that the concentration of Cs and Sr in the sediments is equivalent to that in the suspended solids, as these are sandy sediments with very low concentrations of silt and mud. For this reason, the results obtained are significantly lower than those given in IAEA TRS 472 (IAEA 2010).

**Table 5:** Water–sediment transfer coefficient, apparent *K*_*d*_, calculated in this study and compared with those obtained from field measurements and summarised in the IAEA TRS 472 report

	Water–sediment transfer coefficient *K*_*d*_ (L/Bq)				IAEA TRS 472 *K*_*d*_ (L/Bq) (field measurements)	
	Average ± SD	Median	*N*	Range	GM	Range
^90^Sr	60 ± 30	50	219	12–193	1.2·10^3^	2.3·10^2^–6.3·10^3^
^137^Cs	400 ± 300	340	52	21–1417	2.9·10^4^	1.6·10^3^–5.2·10^5^
^134^Cs	150–30	145	7	84–179		

*N* number of samples, *GM* geometric median

### Dose assessment to humans and non-human biota (carps)

The occurrence of anthropogenic radionuclides in carps may influence de dose to humans by ingestion. The assessment of the effective dose due to carp consumption (Eq. 8) was carried out in the worst-case scenario: all the annual intake of fish was due to muscle carp consumption and all the reported radionuclides were present and at the maximum concentration determined.

8 $${H}_{e}\left(Sv/y\right)={\sum }_{i}{C}_{i}\cdot m\cdot {e}_{i}$$

where *C*_*i*_ is the concentration of radionuclide *i* in muscle carp, expressed in Bq/kg fw; m is the annual consumption of fish in Spain (kg fw/y), estimated in 22.53 kg fw/y (MAPA 2021); and *e*_*i*_ is the dose conversion coefficient for ingestion for adults, expressed in Sv/Bq (IRCP 2012).

The annual effective dose due to carp consumption in this worst-case scenario was 0.020 mSv/y, well below the reference value of 1 mSv/y for the general population (EU 2014). Therefore, the carp consumption posed no radiological hazard to humans.

The assessment of the dose rate to non-human biota (carps) in this freshwater ecosystem was carried out in ERICA Tool 2.0, using Tier 3 in order to obtain a more realistic evaluation. Whole organism activity concentrations were estimated applying the Ratio_wo:muscle_ used in Eq. 1. Whenever possible, experimental transfer parameters (*C*_*R*_ and apparent *K*_*d*_) were used; otherwise, the predefined values in ERICA were used. All distributions (transfer factors, activity concentration in water and sediments) were considered to be log-normal (Brown et al. 2008; Wood et al. 2013). The total dose rate was estimated to be 2.57·10^−4^ µGy/h (6.17 nGy/d) well below the screening value of 10 µGy/h used in ERICA Tool (Brown et al. 2008) and range 1–10 mGy/d for Derived Consideration Reference Levels (DCRL) used by ICRP (ICRP 2014). Therefore, no adverse ecological effects are expected to occur.

**Fig. 5:**
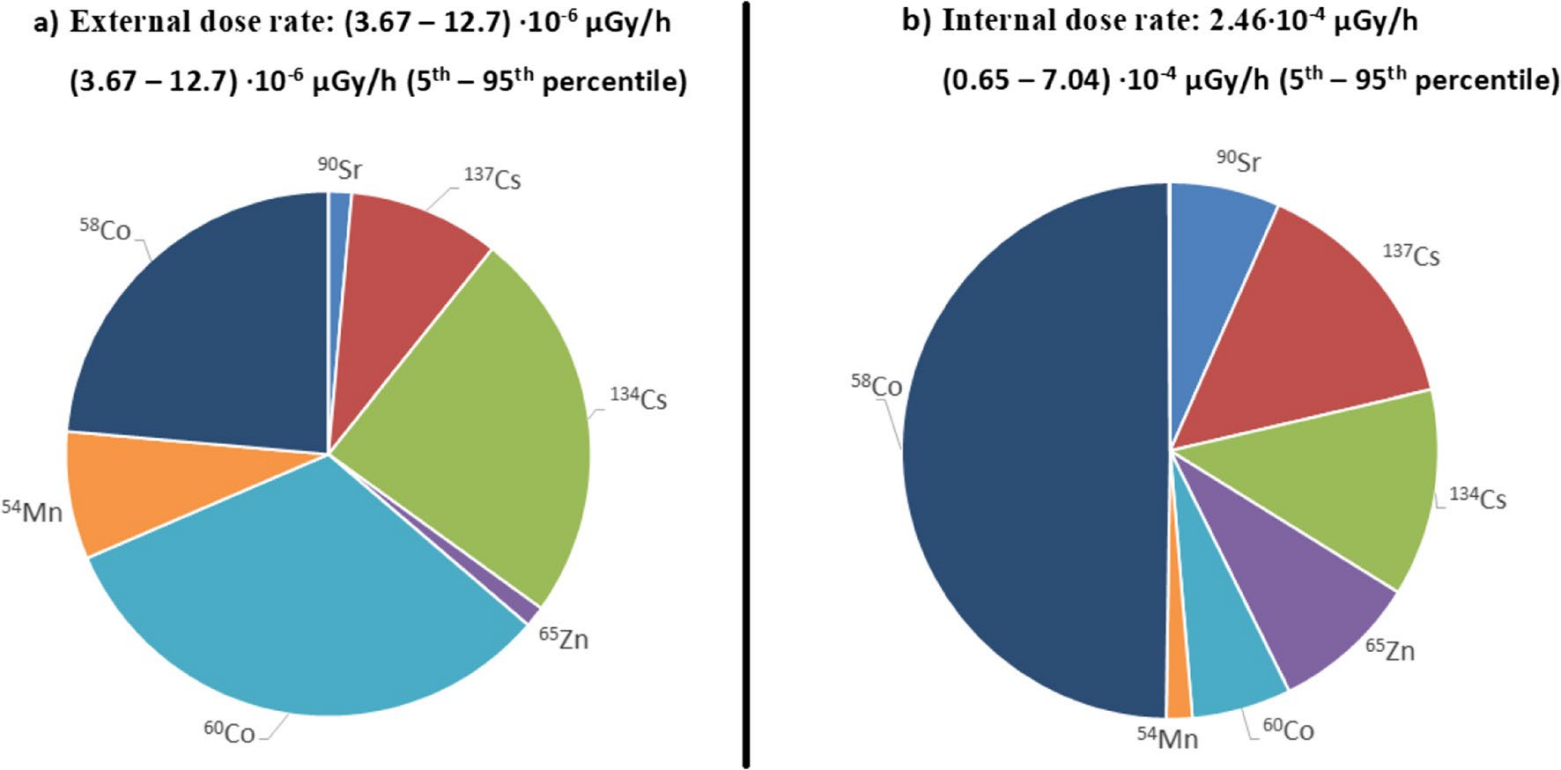
Contribution of the different radionuclides analysed (^90^Sr, ^134,137^Cs, ^65^Zn, ^58,60^Co, and ^54^Mn) to the **a** external and **b** internal dose rates for carps

The total dose rate was mainly due to internal dose rate, which was assessed in 2.46·10^−4^ µGy/h within the range (0.65–7.04) ·10–4 µGy/h (5th^–^95th percentile). The external dose rate was assessed in 7.84·10^−6^ µGy/h within the range (3.67–12.7) ·10^−6^ µGy/h (5th–95th percentile). Figure 5 shows the contribution of the different radionuclides considered to the internal and external dose. The main contributors for external dose rate were ^60^Co, followed by ^134^Cs, ^58^Co, and ^137^Cs, adding up to 89.4% of it, whereas the main contributors for the internal dose rate were ^58^Co (49.8%), followed by ^137^Cs, ^134^Cs (27.2%), ^65^Zn, ^90^Sr, and ^60^Co.

## Conclusions

The long-term concentration of natural and man-made radionuclides in a Mediterranean freshwater ecosystem corresponding to the cooling pond of the ANPP, which is currently in operation, has been assessed in this study. Representative samples of biota (carp, bulrush and cattail), sediments and water were analysed. The levels of activity concentration in the biota samples are consistent with those found in bibliography at similar sites but with very different climates. In addition, transfer coefficients for the main man-made radionuclides detected were computed, and it was found that these were significantly lower than the typical ranges found for benthic fish and vascular plants in freshwater ecosystems.

The following detailed results were obtained:

1. As *K* is a homeostatic element, the concentration levels of ^40^ K remained constant in the biota samples, although a trend was observed in the carp probably due to the variability of temperature changes in the water. Concerning the concentration levels of man-made radionuclides, a decreasing trend of ^90^Sr is observed in all the biota samples, which indicates that the greater presence of this element in the biota is due to the global fallout and, to a much lesser extent, to the discharges of the ANPP. This is not the case for ^137^Cs, which shows changes in concentration levels due to routine discharges.
2. An evaluation of the effective, ecological and environmental half-lives of ^90^Sr and ^137^Cs in biota, sediment and water samples has been carried out, and it has been observed that they do not differ significantly from others obtained in ecosystems with very different climates. Differences in biological removal of ^90^Sr are observed in biota. Thus, for carp this removal is slow, the effective half-life is almost the same as the physical half-life, while for bulrush there is a faster removal rate, which, however, is not observed in cattail.
3. The CR_wo:water_ and CR_wo:sediment_ transfer coefficients were calculated for a Mediterranean freshwater ecosystem. The results obtained show that the values are in line with, but slightly lower than, those tabulated in the IAEA TRS 479 report.
4. The internal and external doses received by the carp exposed to the man-made radionuclides discharges in ANPP’s routine operation have been evaluated using ERICA tool. The result shows that the highest dose is below the screening value of 10 µGy/h used in ERICA Tool and range 1–10 mGy/d for Derived Consideration Reference Levels (DCRL) used by ICRP.

## Data Availability

Data will be made available on request.

## References

[CR1] Aarkrog A, Dahlgaard H, Nielsen SP (2000). Environmental radioactive contamination in Greenland: a 35 years retrospect. Sci Total Environ.

[CR2] AEMET (2011) Iberian Climate Atlas. Air temperature and precipitation (1971–2000) Ministerio de Medio Ambiente, y Medio Rural y Marino. Agencia Estatal de Meteorología. Instituto de Meteorologia de Portugal. ISBN: 978–84–7837–079–5. 10.31978/784-11-002-5

[CR3] AMAP (2010) AMAP Assessment 2009: radioactivity in the arctic. Arctic Monitoring and Assessment Programme (AMAP), Oslo, Norway. xii + 92 pp

[CR4] Brittain JE, Bjørnstad HE (2010). A long-term study of catchment inputs of ^137^ Cs to a subalpine lake in the form of allochthonous terrestrial plant material. Hydrobiologia.

[CR5] Brown JE, Alfonso B, Avila R, Beresford NA, Copplestone D, Pröhl G, Ulanovsky A (2008). The ERICA tool. J Environ Radioact.

[CR6] Brown JE, Alfonso B, Avila R, Beresford NA, Copplestone D, Hosseini A (2016). A new version of the ERICA tool to facilitate impact assessments of radioactivity on wild plants and animals. J Environ Radioact.

[CR7] Ciffroy P, Siclet F, Damois C, Luck M, Duboudin C (2005). A dynamic model for assessing radiological consequences of routine releases in the Loire river: parameterisation and uncertainty/sensitivity analysis. J Environ Radioact.

[CR8] Copplestone DC, Beresford NA, Brown J, Yankovich T (2013). An International database of radionuclide concentration ratios for wildlife: development and uses. J Environ Radioact..

[CR9] Cornett RJ, Ophel IL (1986). Transport of ^60^Co between water and sediments in a small shield lake. Can J Fish Aquat Sci.

[CR10] CSN (2023) https://www.csn.es/kprgisweb2/index.html?lang=es. Last access: October 2023

[CR11] de Cort M, Dubois G, Fridman ShD, Germenchuk MG, Izrael Yu A, Janssens A, Jones AR, Kelly GN, Kvasnikova EV, Matveenko I, Nazarov IM, Pokumeiko Yu M, Sitak VA, Stukin ED, Tabachny LYa, Tsaturov, Yu S, Avdyushin SI (1998) Atlas of caesium deposition on Europe after the Chernobyl accident, Luxembourg, Office for Official Publications of the European Communities, ISBN 92–828–3140-X, Catalogue number CG-NA-16–733–29-C, EUR 16733, 1–63

[CR12] Delistraty D, Van Verst S, Rochette EA (2010). Radiological risk from consuming fish and wildlife to Native Americans on the Hanford Site (USA). Environ Res.

[CR13] Duffa C, Masson M, Gontier G, Claval D, Renaud P (2004). Synthèse des études radioécologiques annuelles menées dans l'environnement des centrales électronucléaires françaises depuis 1991. Radioprotection.

[CR14] Environmental Measurements Laboratory (EML) (1976) Procedures manual HASL-300. US Dept. of Energy, New York

[CR15] EU (2000) Directive 2000/60/EC of the European Parliament and of the Council of 23 October 2000 establishing a framework for Community action in the field of water policy. European Parliament, Council of the European Union. 2000. Official Journal L 327 , 22/12/2000 P. 0001 – 0073

[CR16] EU (2014)Council Directive (2013/59/Euratom) on basic safety standards for protection against the dangers arising from exposure to ionising radiation, and repealing Directives 89/618/Euratom, 90/641/Euratom, 96/29/ Euratom, 97/43/Euratom and 2003/122/Euratom.Off. J. Eur. Union No. L13/1, 17 January 2014 (2014)

[CR17] Felicísimo Pérez AM, Morán López R, Sánchez Guzman JM, Pérez Mayo D (2001) Elaboración del Atlas climático de Extremadura mediante un sistema de información geográfica. GeoFocus (Artículos), nº 1 pp 17–23 in Spanish

[CR18] Franić Z, Marović G (2007). Long-term investigations of radiocaesium activity concentrations in carp in North Croatia after the Chernobyl accident. J Environ Radioact.

[CR19] Gascó C, Alvarez G (1988) Some analytical aspects about determination of Sr 89 and Sr 90 in environmental samples (No. CIEMAT--617). Junta de Energía Nuclear

[CR20] Guillén J, Beresford NA, Baeza A, Izquierdo M, Wood MD, Salas A, Muñoz-Serrano A, Corrales-Vázquez JM, Muñoz-Muñoz JG (2018). Transfer parameters for ICRP’s reference animals and plants in a terrestrial Mediterranean ecosystem. J Environ Radioact.

[CR21] Hanslík E, Jedináková-Křížová V, Ivanovová D, Kalinová E, Sedlářová B, Šimonek P (2005). Observed half-lives of 3H, 90Sr and 137Cs in hydrosphere in the Vltava River basin (Bohemia). J Environ Radioact.

[CR22] Hanslík E, Marešová D, Juranová E, Sedlářová B (2018). Kinetics of ^3^H, ^90^Sr and ^137^Cs content changes in hydrosphere in the Vltava River system (Czech Republic). J Environ Radioact.

[CR23] Hosseini A, Beresford NA, Brown JE, Jones DG, Phaneuf M, Thørring H, Yankovich T (2010). Background dose-rates to reference animals and plants arising from exposure to naturally occurring radionuclides in aquatic environments. J Radiol Prot.

[CR24] Howard BJ, Beresford NA, Copplestone D, Telleria D, Proehl G, Fesenko S, Jeffree R, Yankovich T, Brown J, Higley K, Johansen M, Mulye H, Vandenhove H, Gashchak S, Wood MD, Takata H, Andersson P, Dale P, Ryan J, Bollh€ofer A, Doering C, Barnett CL, Wells C (2013). The IAEA handbook on radionuclide transfer to wildlife. J Environ Radioact..

[CR25] International Atomic Energy Agency (IAEA) (2010) Handbook of parameter values for the prediction of radionuclide transfer in terrestrial and freshwater environments. Technical reports series No. 472. IAEA, Vienna

[CR26] International Atomic Energy Agency (IAEA) (2014) Handbook of parameter values for the prediction of radionuclide transfer to wildlife. Technical Reports Series No. 479. IAEA, Vienna

[CR27] International Commission on Radiological Protection (ICRP) (1991) The 1990 recommendations of the international commission on radiological protection. ICRP Publication 60. Ann ICRP 21(1–3) 2053748

[CR28] International Commission on Radiological Protection (ICRP) (1997) Recommendations of the international commission on radiological protection. ICRP Publication 26. Ann ICRP 1(3) 10.1016/j.icrp.2007.10.003 18082557

[CR29] International Commission on Radiological Protection (ICRP) (2008a) Nuclear decay data for dosimetric calculations. ICRP Publication 107. Ann ICRP 38(3) 10.1016/j.icrp.2008.10.004 19285593

[CR30] International Commission on Radiological Protection (ICRP) (2008b) Environmental protection - the concept and use of reference animals and plants. ICRP Publication 108. Ann ICRP 38(4–6)

[CR31] International Commission on Radiological Protection (ICRP) (2009) Environmental protection: transfer parameters for reference animals and plants. ICRP Publication 114. Ann ICRP 39(6) 10.1016/j.icrp.2011.08.009 22108188

[CR32] International Commission on Radiological Protection (ICRP) (2014) Protection of the environment under different exposure situations. ICRP Publication 124. Ann ICRP 43(1) 10.1177/0146645313497456 25915706

[CR33] International Organization for Standarization (2017) ISO/IEC 17025:2017. General requirements for the competence of testing and calibration laboratories. Geneva

[CR34] Jannik GT, Baker RA, Lee PL, Eddy TP, Blount GC, Whitney GR (2013) Long-term assessment of critical radionuclides and associated environmental media at the Savannah River Site–13038. In Wildlife Management Conference

[CR35] Janovics R, Bihari Á, Papp L, Dezső Z, Major Z, Sárkány KE, ... Palcsu L (2014) Monitoring of tritium, ^60^Co and ^137^Cs in the vicinity of the warm water outlet of The Paks Nuclear Power Plant, Hungary. J Environ Radioact 128:20–26. 10.1016/j.jenvrad.2013.10.023 10.1016/j.jenvrad.2013.10.023 24246753

[CR36] Kottek M, Grieser J, Beck C, Rudolf B, Rubel F (2006). World map of the Köppen- Geiger climate classification updated. Meteorol Z.

[CR37] Ministerio de Agricultura, Pesca y Alimentación (MAPA) (2021) Informe del consumo de alimentación en España 2020. Ministerio de Agricultura, Pesca y Alimentación. Madrid (In spanish)

[CR38] McCreedy CD, Jagoe CH, Glickman LT, Brisbin IL (1997). Bioaccumulation of cesium-137 in yellow bullhead catfish (Ameiurus natalis) inhabiting an abandoned nuclear reactor reservoir. Environ Toxicol Chem.

[CR39] Metz JR, van den Burg EH, Bonga SEW, Flik G (2003). Regulation of branchial Na+/K+-ATPase in common carp Cyprinus carpio L. acclimated to different temperatures. J Exp Biol.

[CR40] Nedveckaite T, Filistovic V, Marciulioniene D, Prokoptchuk N, Plukiene R, Gudelis A, ... Beresford NA (2011) Background and anthropogenic radionuclide derived dose rates to freshwater ecosystem–Nuclear power plant cooling pond–Reference organisms. J Environ Radioact 102(8):788–795. 10.1016/j.jenvrad.2011.04.012 10.1016/j.jenvrad.2011.04.012 21601320

[CR41] Noshkin VE, Robison WL, Wong KM, Brunk JL, Eagles RJ, Jones HE (1997). Past and present levels of some radionuclides in fish from Bikini and Enewetak Atolls. Health Phys.

[CR42] OpenStreetMap (2015) OpenStreetMap contributors. Planet dump. Retrieved from https://planet.openstreetmap.org. Last access: October 2023

[CR43] Paller MH, Jannik GT, Baker RA (2014). Effective half-life of caesium-137 in various environmental media at the Savannah River Site. J Environ Radioact.

[CR44] Proehl G, Fiedler I, Klemt E, Zibold G, Ehlken S (2004) Recording of ecological half-lives of ^90^Sr and ^137^Cs in terrestrial and aquatic ecosystems; Erfassung oekologischer Halbwertszeiten von ^90^Sr und ^137^Cs in terrestrischen und aquatischen Oekosystemen. Germany

[CR45] Rowan DJ, Rasmussen JB (1994). Bioaccumulation of radiocesium by fish: the influence of physicochemical factors and trophic structure. Can J Fish Aquat Sci.

[CR46] Smith JT, Sasina NV, Kryshev AI, Belova NV, Kudelsky AV (2009). A review and test of predictive models for the bioaccumulation of radiostrontium in fish. J Environ Radioact.

[CR47] Smith JT, Beresford NA (2005) Chernobyl ą catastrophe and consequences. ISBN 3–540–23866–2 Springer-Verlag Berlin Heidelberg New York

[CR48] Sundbom M, Meili M, Andersson E, Östlund M, Broberg A (2003). Long-term dynamics of Chernobyl ^137^Cs in freshwater fish: quantifying the effect of body size and trophic level. J Appl Ecol.

[CR49] Tagami K, Uchida S (2016). Consideration on the long ecological half-life component of ^137^Cs in Demersal fish based on field observation results obtained after the Fukushima accident. Environ Sci Technol.

[CR50] Tjahaja PI, Sukmabuana P, Salami IRS, Muntalif BS (2012). Laboratory experiment on the determination of radiostrontium transfer parameter in water–fish compartment system. J Environ Radioact.

[CR51] Wada T, Nemoto Y, Shimamura S, Fujita T, Mizuno T, Sohtome T, ... Igarashi S (2013) Effects of the nuclear disaster on marine products in Fukushima. J Environ Radioact 124:246–254. 10.1016/j.jenvrad.2013.05.008 10.1016/j.jenvrad.2013.05.008 23831549

[CR52] Wood MD, Beresford NA, Howard BJ, Copplestone D (2013). Evaluating summarised radionuclide concentration ratio datasets for wildlife. J Environ Radioact.

[CR53] Yankovich TL, Beresford NA, Wood MD, Aono T, Andersson P, Barnett CL, ... Uchida S (2010) Whole-body to tissue concentration ratios for use in biota dose assessments for animals. Radiat Environ Biophys 49:549–565. 10.1007/s00411-010-0323-z 10.1007/s00411-010-0323-z 20931337

